# Protective effect of house screening against indoor *Aedes aegypti* in Mérida, Mexico: A cluster randomised controlled trial

**DOI:** 10.1111/tmi.13680

**Published:** 2021-10-21

**Authors:** Pablo Manrique‐Saide, Josué Herrera‐Bojórquez, Josué Villegas‐Chim, Henry Puerta‐Guardo, Guadalupe Ayora‐Talavera, Manuel Parra‐Cardeña, Anuar Medina‐Barreiro, Marypaz Ramírez‐Medina, Aylin Chi‐Ku, Emilio Trujillo‐Peña, Rosa E. Méndez‐Vales, Hugo Delfín‐González, María E. Toledo‐Romaní, Roberto Bazzani, Edgardo Bolio‐Arceo, Hector Gómez‐Dantés, Azael Che‐Mendoza, Norma Pavía‐Ruz, Oscar D. Kirstein, Gonzalo M. Vazquez‐Prokopec

**Affiliations:** ^1^ Unidad Colaborativa para Bioensayos Entomológicos Campus de Ciencias Biológicas y Agropecuarias Universidad Autónoma de Yucatán Mérida México; ^2^ Laboratorio de Virología Centro de Investigaciones Regionales ‘Dr. Hideyo Noguchi’ Universidad Autónoma de Yucatán Mérida México; ^3^ Servicios de Salud de Yucatán Mérida México; ^4^ Departamento de Epidemiología Instituto de Medicina Tropical ‘Pedro Kourí’ La Habana Cuba; ^5^ International Development Research Centre of Canada Regional Office for Latin America and the Caribbean Montevideo Uruguay; ^6^ Instituto Municipal de Planeación Ayuntamiento de Mérida Mérida México; ^7^ Centro de Investigación en Sistemas de Salud Instituto Nacional de Salud Pública Cuernavaca México; ^8^ Laboratorio de Hematología Centro de Investigaciones Regionales ‘Dr. Hideyo Noguchi’ Universidad Autónoma de Yucatán Mérida México; ^9^ Department of Environmental Sciences Emory University Atlanta Georgia USA

**Keywords:** *Aedes aegypti*, *Aedes*‐transmitted viruses, arboviruses, House screening, Merida

## Abstract

**Objective:**

To evaluate the protective effect of house screening (HS) on indoor *Aedes aegypti* infestation, abundance and arboviral infection in Merida, Mexico.

**Methods:**

In 2019, we performed a cluster randomised controlled trial (6 control and 6 intervention areas: 100 households/area). Intervention clusters received permanently fixed fiberglass HS on all windows and doors. The study included two cross‐sectional entomologic surveys, one baseline (dry season in May 2019) and one post‐intervention (PI, rainy season between September and October 2019). The presence and number of indoor *Aedes* females and blood‐fed females (indoor mosquito infestation) as well as arboviral infections with dengue (DENV) and Zika (ZIKV) viruses were evaluated in a subsample of 30 houses within each cluster.

**Results:**

HS houses had significantly lower risk for having *Aedes aegypti* female mosquitoes (odds ratio [OR] = 0.56, 95% CI 0.33–0.97, *p *= 0.04) and blood‐fed females (OR = 0.53, 95% CI 0.28–0.97, *p *= 0.04) than unscreened households from the control arm. Compared to control houses, HS houses had significantly lower indoor *Ae*. *aegypti* abundance (rate ratio [RR] = 0.50, 95% CI 0.30–0.83, *p *= 0.01), blood‐fed *Ae*. *aegypti* females (RR = 0.48, 95% CI 0.27–0.85, *p *= 0.01) and female *Ae*. *aegypti* positive for arboviruses (OR = 0.29, 95% CI 0.10–0.86, *p *= 0.02). The estimated intervention efficacy in reducing *Ae*. *aegypti* arbovirus infection was 71%.

**Conclusions:**

These results provide evidence supporting the use of HS as an effective pesticide‐free method to control house infestations with *Aedes aegypti* and reduce the transmission of *Aedes*‐transmitted viruses such as DENV, chikungunya (CHIKV) and ZIKV.

## INTRODUCTION

Arboviral diseases caused by *Aedes*‐transmitted viruses (ATV) such as DENV, CHIKV and ZIKV present a significant public health problem in urban areas worldwide. The widespread distribution of *Ae*. *aegypti*, the main ATV vector in the Americas, puts approximately 500 million people at risk of dengue infection [1] and has fuelled the pandemic propagation of novel viruses such as chikungunya [2] and Zika [3,70,71]. Without commercially efficacious and fully licensed vaccines or therapeutics available against many of these arboviral infections, vector control aimed at reducing mosquito vector populations and/or their contact with humans remains the immediate alternative to reduce or prevent ATV transmission [6]. Current vector control strategies of ministries of health (MoH) primarily focus on reducing vector density by either targeting immature stages and their habitats or the adult mosquito population [6]. Although interventions to reduce vector contact with humans are routinely recommended, personal protection and modifications/improvements to the built environment (e.g. mosquito proofing of houses) are seldom implemented as part of MoH programmes.

House screening (HS), covering doors and windows with mosquito nets/screens, is a house improvement and a pesticide‐free control approach to reduce human–*Ae*. *aegypti* contacts [7, 8]. For decades, people had used netting of different materials to screen their houses and prevent the entry of nuisance (or disease‐carrying) insects [9,10]. This approach is particularly prevalent in urban areas, as building structure and economic resources facilitate their adoption. While HS is identified as an example of a housing intervention following the principle of ‘Keeping the vector out’ promoted by WHO [6, 11], this intervention has been largely overlooked by policies and programmes for the prevention and control of ATVs [12, 13]. It was not until 2017 that the WHO Special Programme for Research and Training in Tropical Diseases (TDR) cited HS as a promising vector management approach for the prevention and control of ATVs [14]. Recent meta‐analyses and systematic reviews provide evidence of the effectiveness of house screens on external doors and windows in preventing dengue transmission [15, 16]. However, stronger evidence of its efficacy obtained from field randomised trials is recognised as necessary.

In the last decade, projects within the ‘Eco‐Bio‐social Research’ and ‘Ecohealth’ programmes in Mexico supported by TDR and the International Development Research Centre (IDRC) showed that insecticide‐treated screening (ITS, long‐lasting insecticide‐treated nets fixed with aluminium frames on doors and windows) acts as a physical/chemical barrier that confers sustained protection against indoor female *Aedes aegypti* infestation [17, 18, 19, 20, 21]. Moreover, ZIKV detection in *Ae*. *aegypti* during a Zika outbreak was reduced by 85% in clusters with ITS versus untreated control clusters [21]. Although ITS is a widely accepted intervention by the community [18, 22], its accessibility is limited because insecticide‐treated nets (ITNs) are not yet commercially available for public use since they are exclusively sold to the Ministry of Health in Mexico [23]. Given that the insecticidal effect of LLINs wanes after a couple of years [16], the sustainability of both HS and LLINs depends on careful evaluations of their cost, scalability, and entomological/epidemiological impacts.

The main goal of this study was to evaluate, in an entomological cluster randomised control trial (CRCT), the efficacy of screening doors and windows with a regular mosquito mesh in reducing infestation with, abundance of and infection by indoor collected *Ae*. *aegypti* mosquitoes in the Mexican city of Merida, Yucatan, in 2019. We also assessed the domestic practices implemented by the study participants to reduce mosquitoes and mosquito‐borne diseases, as well as the perception and acceptance for HS in intervened households. We hypothesised that by reducing the abundance of *Ae*. *aegypti* inside households using HS, a reduction in infection in *Ae*. *aegypti* can be achieved.

## MATERIAL AND METHODS

### Study site

Merida (20°58′2.532″N; 89°35′33.3096″W) is the capital and the major urban centre of the state of Yucatan, with a population of 921,771 inhabitants living in 284,468 households [24]. Average elevation of the city is 9 metres above sea level and the climate is mainly warm with an annual average temperature of 26–27°C (36°C max to 18°C min). Although there is continuous dengue virus (DENV) transmission throughout the year, two distinct seasons can be clearly identified: a rainy season from May to October and a dry season from November to April. The rainy season is historically associated with mosquito abundance, dengue transmission (increases 80%) and augmented vector control activities [25].

At the national level, Merida is among the cities that have reported the highest proportion of dengue cases in the last 15 years (2.6%) and accounted for >40% of all dengue cases in the state of Yucatan during the last decade [26]. The first cases of chikungunya in Merida and a subsequent outbreak (1531 cases) occurred in 2015 and transmission decreased during the following years (11 cases in 2016, and 0 cases in 2017–2018) [26, 27]. ZIKV transmission was initially detected in May 2016 with 2,199 cases reported, although transmission decreased to 24 cases in 2017 and 28 cases in 2018 [26, 27]. No laboratory‐confirmed cases of chikungunya and Zika virus were reported during 2019–2020 [28]. Various neighbourhoods in Merida have been historically identified as hotspots because they produce more cases and consistently demand vector control activities [26, 27, 29, 30, 31]. Previous studies in Merida showed that the most important productive container types for *Ae*. *aegypti* immatures are disposable containers, buckets/pots and other rain‐filled objects left in backyards [30, 32, 33] along with non‐residential habitats, such as subsurface catch basins (e.g. drainage systems, storm drains, street drainage) [31, 32].

### Experimental design

The study followed a standard two‐arm entomological CRCT design, comparing six clusters with the intervention (HS) with another six clusters without HS (as control) during the peak of mosquito abundance, corresponding to the rainy season [18, 21, 25]. As in previous studies [17, 18, 20, 21], we originally planned to carry out the post‐intervention evaluation for a second year, but this activity was halted by the COVID‐19 pandemic.

**FIGURE 1:**
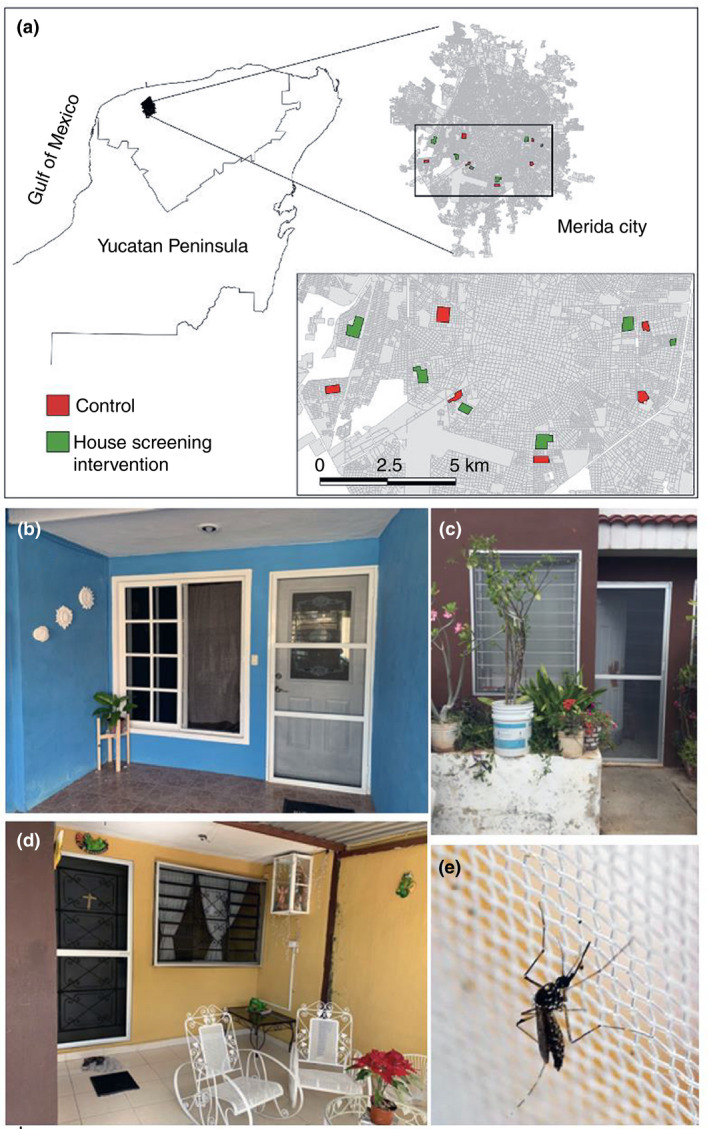
(a) Study site showing the location of the study areas and randomly selected clusters with and without house‐screening (HS) interventions in the city of Merida, Mexico. (b–d) House screening – with regular netting mounted on aluminium frames – installed on doors and windows of houses. (e) Female *Aedes aegypti* standing on a screen

Twelve clusters comprising 100 households each (1200 houses in total) in different neighbourhoods of Merida (*n* = 12) were selected based on their entomological and epidemiological importance according to the local vector control programme (Figure 1a). These 12 clusters were numerically and blindly randomised using an Excel spreadsheet (MS Excel 365, 2019) to generate two groups of six clusters each. On a second round of randomisation, one group was selected to receive the intervention (*n* = 6) and the other group remained as control (*n* = 6) (Figure 1, Figure S1). The clusters comprised an average set of 18 city blocks (each block had, on average, 25 premises) located within the areas previously identified as hotspots of *Aedes*‐borne virus transmission [27]. Clusters localisation comprised residential areas, where about 23,330 inhabitants live [24]. Entomological evaluations were conducted on a random sample of 30 houses per cluster (intervention: 180 houses; control: 180 houses) (Figure S1). Not all premises within a block were enrolled in the study because they were small businesses, empty, or householders who declined to participate or were absent at the time of enrolment. Houses included in the study were typically single storey, made of cement‐plastered blocks with a closed roof and with no ventilating features (e.g. ventilation bricks, eaves, etc.) other than windows (Figure 1b; Table S1).

We powered the study to detect a significant difference in our primary entomological endpoint: the density of *Ae aegypti* indoors collected after a 10‐min Prokopack aspiration session [21]. Based on an expected effect size of 70% in the reduction in *Ae*. *aegypti* indoors by ITS [18] from an expected mean baseline number of 4.4 ± 9 [36], an alpha of 0.05 and a power of 80%, we estimated a total of 134 houses per arm (268 total houses) to detect a significant difference between groups (https://clincalc.com/stats/samplesize.aspx). Therefore, our trial size provided enough statistical power to evaluate a difference at even a lower effect size than 60%.

### House screening

The installation of HS ran from July to August 2019. Regular fiberglass net (brand Herralum®, available in 30 m length x 1.50 m width rolls, colour grey, mesh light 0.6 x.07 mm and density 0.32mm) was mounted in aluminium frames custom fitted to doors and windows of houses (Figure 1b–d) in collaboration with a third‐party local small business (Vidrios y aluminios Bojorquez S.A) as described in Manrique‐Saide et al. [21, 31] and Che‐Mendoza et al. 2015, 2018, [17, 18]. During the installation, at least one person in every household received information about the proper use and maintenance of HS from the research staff. The main recommendation to the householder to keep the door closed as much as possible. The average cost of the HS per house was US $141.66. This average price included screening of two doors and seven windows of a typical 75 m^2^ household (floor area) (Figure 1b–e).

Both areas received routine vector control as part of national policy in response to dengue outbreaks and entomological risk indices [37]. The activities during 2019 included: outdoor spraying with organophosphates (malathion), fast‐acting pyrethroids (transfluthrin) and neonicotinoids plus pyrethroids (imidacloprid +prallethrin); and indoor space spraying with carbamates (propoxur and bendiocarb) and larviciding with spinosyns, bacterial insecticides (Bti), insect growth regulators (methoprene and pyriproxyfen) and organophosphates (pirimiphos‐methyl and temephos).

### Entomological studies

Two cross‐sectional entomological surveys were conducted in intervention and control clusters as in Manrique‐Saide et al. [21, 31] and Che‐Mendoza et al. 2015, 2018 [17, 18]. Indoor adult mosquito collections were performed in a randomly selected subsample of 30 houses from each cluster. From a list of participating houses ordered numerically in each cluster, random numbers were generated until the 30 houses were completed (Figure S1). The baseline survey was completed in May 2019 and was followed by a post‐intervention (PI) survey during the wet season (September to October) in 2019. The primary outcome measures were indoor *Aedes* mosquito density and *Aedes aegypti* infection with *Aedes*‐borne viruses.

Indoor adult mosquitoes were collected with Prokopack aspirators [38] during a 10‐min period per house. Collections within each cluster were performed by three teams of two skilled collectors each on the same day between 09:00 and 12:00 hrs. Considering HS from our study is easily recognisable, entomological collections could not be blinded to the intervention. All mosquitoes collected were identified to species and sex and stored for molecular detection of viral infection.

### Detection of DENV and ZIKV infection in *Aedes* mosquitoes

The study included the detection of DENV and ZIKV genome in female *Ae*. *aegypti* collected from the same sample of houses [*n* = 183 houses divided into HS (*n* = 80 houses) and control (*n* = 103 houses)], in which we performed the entomological collections for baseline and post‐intervention surveys.

A total of 194 pools (1 to 6 mosquitoes per pool) of field‐collected female *Aedes* mosquitoes were preserved in Eppendorf tubes containing RNA stabilisation reagent (RNAlater; Thermo Scientific). Samples were initially stored at −20°C at the Collaborative Unit for Entomological Bioassay (UADY), then transported to the Virology Laboratory of the Regional Research Center ‘Dr. Hideyo Noguchi’ (CIR‐UADY) for further analysis. Pools were processed for RNA extraction followed by molecular detection of viral RNA genome using an in‐house endpoint RT‐PCR assay. Briefly, each pool of female *Ae*. *aegypti* mosquitoes was initially disinfected with 70% ethanol at room temperature for 2 h. Then, samples were mechanically homogenised in 150 μl of sterile PBS1X using a sterile pestle and electric homogeniser as previously described [39]. RNA extraction was performed using a commercial QIAamp Viral RNA Mini kit (QIAGEN) following the manufacturer's instructions. RNA extract was eluted in nuclease‐free water (Ambion) and quantified using a nanodrop (Thermo Scientific). Finally, extracts were stored at −80°C until further analyses.

DENV and ZIKV infections in *Ae*. *aegypti* mosquitoes were examined by an end‐point one‐step RT‐PCR. Primers were designed to target a ~200 bp fragment of the viral gene NS5 of DENV (DENV‐F: ACAAGTCGAACAACCTGGTCCAT; DENV‐R: GCCGCACCATTGGTCTTCTC) [40], or a fragment of ~100 bp of the viral E gene of ZIKV (ZIKV‐F: CCGCTGCCCAACACAAG; ZIKV‐R: CCACTAACGTTCTTTTGCAGACAT) [41]. The RT‐PCR protocol was performed using a Mastercycler EP Gradient‐Thermal‐Cycler (Eppendorf) and the OneStep RT‐PCR Kit with a master mix including the following components: QIAGEN OneStep RT‐PCR Buffer (5×), dNTP Mix (10 mM each), QIAGEN OneStep RT‐PCR Enzyme Mix, Q‐solution (5×), forward and reverse primers (10 µM), RNAse free‐water and extracted RNA template (100–200 ng per reaction). Amplification parameters were established as follows: initial reverse transcription step at 50°C for 30 min, followed by an initial PCR activation step at 95°C for 15 min and 40 cycles of denaturation at 95°C for 1 min, Tm annealing at 53°C for 1 min and extension at 72°C for 1 min; and final extension at 72°C for 5 min. Viral RNA extracted from DENV and ZIKV strains grown in C6/36 cells (*Ae*. *albopictus*, from the CDC [USA]) were used as positive controls. RNA extracted from a laboratory‐reared *Aedes aegypti* strain from Yucatan was used as negative control. Amplicons were visualised using agarose gel (1.5%) stained with Syber safe (Thermo Scientific) under UV excitation. Screening for arboviral infections was blindly performed at C.I.R‐UADY.

### Social assessment

As in previous studies, our team performed a social assessment focused on the community initial response during the enrolment (pre‐intervention) and a post‐intervention acceptance and perceived efficacy survey among the participants [21, 22]. In February–March 2019 (during the enrolment process and before the intervention), face‐to‐face household surveys were conducted among 150 heads of family randomly selected from houses in intervened clusters to address the social response of the project. Topics included knowledge, attitudes and practices (KAP survey) on mosquito‐borne diseases as well as personal and domestic preventive measures.

In June 2020 (post‐intervention), a second household survey was applied to 100 family heads interviewed during the enrolment to evaluate the social acceptance and the perceived efficacy of the intervention. Topics explored were the acceptance of intervention, opinion on the installation process, perception of temperature increase associated with HS, perceived reduction in mosquitoes inside the houses, positive cases of DEN/CHIK/ZIK reported by the families after the installation of the mosquito screens and recommendations for scaling‐up the HS method. Because of the COVID‐19 contingency, the questionnaires were applied through telephone calls to guarantee the safety of participants and the scientific team.

### Data analysis

From indoor Prokopack adult collections, we calculated: (a) Houses positive to at least one female *Ae*. *aegypti* (%); (b) Houses positive to blood‐fed female *Ae*. *aegypti* (%); (c) Number of females per house and (d) Number of total blood‐fed females per house. We also report the prevalence of positive houses to indoor female *Ae*. *aegypti* with arbovirus infection (houses with at least one pool of *Ae*. *aegypti* females positive to the presence of arboviral RNA genome [e.g. DENV and ZIKV]).

Logistic regression models (for presence–absence mosquito data) and negative binomial models (for count data) accounting for each house's cluster (cluster‐robust SE calculation) were performed for each cross‐sectional entomological evaluation survey. Odds ratios (OR) and rate ratios (RR) with 95% CI were assessed and significance expressed at the 5% level. Analyses were performed using STATA 13.0 (Stata Corp, College Station, TX, USA).

Values from the infection calculation were used to estimate a measure of epidemiological efficacy, as HSeff = (1−OR) × 100 [42]. This value ranks between 0 and 100 and indicates the proportional reduction in *Ae*. *aegypti* infection in the intervention arm compared to the control arm.

### Ethics statement

This study was approved by the ethical committee of CCBA‐UADY (CB‐CCBA‐I‐2019–003). Written informed consent was obtained for each participating household (householder over the age of 18) at the beginning of the study.

## RESULTS

### Impact of HS on indoor adult mosquitoes

A total of 897 adult indoor resting mosquitoes (413 males, 484 females) were collected during the whole study period. *Ae*. *aegypti* was the most abundant species, representing 76% of the total collection (682 [320males, 362 females]), followed by *Culex* spp. (23%, 206/897) and a few *Ochlerotatus taeniorhynchus* (1%, 9/897).

Entomological indicators are summarised on Table 1. During the pre‐intervention survey (dry season, May to June 2019), adult‐based entomological indicators showed similar seasonal patterns of house infestation in both study arms (Table 1). Indoor *Ae*. *aegypti* females at different feeding stages were collected among 20–30% (36–54/180) houses in both study arms.

**TABLE 1:** Entomological indicators for control and HS intervention surveys during dry and rainy seasons

Survey	Treatment	Mean	SEM	OR/IRR	95% CI	*p* value
Houses positive for *Aedes* females						
Dry season 2019	Control	0.27	0.03			
	HS intervention	0.24	0.03	0.86	0.47–1.58	0.64
Rainy season 2019	Control	0.30	0.03			
	HS intervention	0.19	0.03	**0.56**	**0.33–0.97**	0.04*
Houses positive for blood‐fed *Aedes* females						
Dry season 2019	Control	0.27	0.03			
	HS intervention	0.23	0.03	0.81	0.45–1.45	0.48
Rainy season 2019	Control	0.28	0.03			
	HS intervention	0.17	0.03	**0.53**	**0.28–0.97**	0.04*
Number of female *Aedes* per house						
Dry season 2019	Control	0.41	0.07			
	HS intervention	0.57	0.10	1.41	0.71–2.79	0.32
Rainy season 2019	Control	0.69	0.12			
	HS intervention	0.34	0.06	**0.50**	**0.30–0.83**	0.01*
Number of blood‐fed female *Aedes* per house						
Dry season 2019	Control	0.39	0.07			
	HS intervention	0.51	0.1	1.30	0.67–2.51	0.44
Rainy season 2019	Control	0.65	0.12			
	HS intervention	0.31	0.06	**0.48**	**0.27–0.85**	0.01*
House positive for *Aedes* females infected with arboviruses (pools)						
Dry season 2019	Control	0.18	0.03			
	HS intervention	0.11	0.02	0.55	0.19–1.58	0.27
Rainy season 2019	Control	0.20	0.03			
	HS intervention	0.07	0.02	**0.29**	**0.1–0.86**	0.025*
House positive for infected *Aedes* DENV (pools)						
Dry season 2019	Control	0.13	0.02			
	HS intervention	0.08	0.02	0.58	0.19–1.71	0.32
Rainy season 2019	Control	0.19	0.03			
	HS intervention	0.06	0.02	**0.28**	**0.09–0.85**	0.024*
House positive for infected *Aedes* ZIKV (pools)						
Dry season 2019	Control	0.14	0.03			
	HS intervention	0.07	0.02	0.42	0.17–1.08	0.07
Rainy season 2019	Control	0.17	0.03			
	HS intervention	0.06	0.02	**0.28**	**0.09–0.91**	0.034*

Note

Comparison between intervened‐treated (HS) and untreated (control) arms on indoor female *Aedes*‐based entomological indicators (*n* = 180 houses per arm) in Merida, Mexico. Odds ratios (OR) and rate ratios (RR) with 95% confidence intervals are showed for presence–absence data and count data, respectively, for each cross‐sectional entomological survey by arm. * Statistical significance is indicated in bold (*p* < 0.05).

Abbreviation: HS, house screening.

After the intervention (rainy season 2019), adult *Ae*. *aegypti* abundance was significantly lower in the houses protected with HS than in the houses not protected with HS (Table 1). Houses with HS had significantly lower risk of having *Ae*. *aegypti* female mosquitoes (OR = 0.56, 95% CI 0.33–0.99) and blood‐fed females (OR = 0.53, 95% CI 0.28–0.97) in comparison with unscreened households. Indoor abundance of *Ae*. *aegypti* also showed significantly fewer adult females in houses protected with HS (RR = 0.50, 95% CI 0.30–0.83) and fewer blood‐fed females indoors (RR = 0.48, 95% CI 0.27–0.85) (Table 1).

### Impact of HS in houses with pools of female *Ae. aegypti* positive for arbovirus

Among 360 houses from both arms sampled during the study, a total of 26% (93/360) and 25% (89/360) were positive for *Ae*. *aegypti* females during the dry and rainy season respectively. A total of 194 female *Ae*. *aegypti* pools (mean of 1.06/house positive to females) were analysed for DEN/ZIK infection. A total of 99/194 pools (51%) were positive for arboviruses, from which specifically 42% (82/194) and 40% (79/194) were positive for DENV and ZIKV respectively.

At baseline (dry season), no significant differences were observed between study arms on the prevalence of arbovirus‐positive pools (Table 1). After HS implementation, having screens was significantly associated with fewer houses with indoor female *Ae*. *aegypti* positive for either arbovirus (OR = 0.29, 95% CI 0.10–0.86, *p *= 0.02). Although we continued detecting indoor *Ae*. *aegypti* females with DENV and ZIKV in houses from both study arms, the proportion of houses with HS positive for *Ae*. *aegypti* females with arbovirus was lower (7%) than in unprotected houses (20%). Based on these data, the estimated intervention effectiveness of HS in reducing arbovirus infection in *Ae*. *aegypti* was HS_eff_ = 71%.

### Knowledge of ATV and preventive practices

The demographic characteristic of surveyed population is included in the supplementary material (Table S2). Participants were already familiar with HS, although none of the houses had HS installed prior the intervention, mainly because of the cost (70%, 105/150), a perceived difficulty for its maintenance (20%, 30/150) and because they could move to another house (10%, 15/150).

Most respondents associated mosquito bites with the infection/transmission of DENV (91%, 134/150), CHIKV (88%, 129/150) and ZIKV (88%, 129/150). They were aware of some clinical manifestations, which were cited as differentially associated with each disease (Table S3). For example, fever was perceived as the main symptom of DENV but not for CHIKV and ZIKV, while joint pain was the most mentioned symptom associated with CHIKV and ZIKV; however, nobody mentioned that ZIKV could be asymptomatic, and respondents were overall less aware about this disease.

Regarding preventive practices, about half of householders reported the use of topical repellents (49%, 71/150) and commercially available insecticide products (68%, 100/150) as the main domestic preventive measures to avoid mosquitoes indoors. The main reason reported by repellents users was the efficacy of the product, while the non‐users said that they could not afford the products. People also used commercially available household insecticides because their perceived efficacy but some people did not use them due to health‐related concerns, for example, having asthmatic relatives at home and the perceived toxicity of the product.

### Social acceptance and perceived efficacy of HS

All interviewed participants reported acceptance of the intervention (HS), high expectations on its efficacy and recommended the scaling‐up of the intervention to other areas of the city. The main reasons for acceptance were to avoid mosquitoes at home (77%, 77/100), concerns about ATV (63%, 63/100) and the free cost of the intervention (54%, 54/100) (Table 2). The majority (94%, 94/100) did not recall having any family member sick from any ATV at home after the installation of HS and most of them (92%, 92/100) believed that HS helped prevent their families from mosquitoes‐borne diseases.

**TABLE 2:** Reasons for acceptability and the perceived efficacy of house screening among the participants of the study

Topics addressed	*N* = 100
Reasons for acceptance of house screening	
To avoid mosquitoes at home	77% (*n* = 77)
Concerns that *Aedes*‐borne diseases could impact their families	63% (*n* = 63)
The free cost of the intervention	54% (*n* = 54)
Impact perceived	
Reduction in mosquitoes indoors after the intervention	
No mosquitoes indoors	66% (*n* = 66)
Reduced number of mosquitoes	29% (*n* = 29)
No reduction in mosquitoes indoors	5% (*n* = 5)
Cases of DEN/CHIK/ZIK reported by the families after the intervention	
No	94% (*n* = 94)
Yes	6% (*n* = 6)
Perception of temperature increase due to house screening	
Did not acknowledge any increase in indoor temperature	80% (*n* = 80)
A light overheating was reported but associated with specific day‐hours (mid‐day)	18% (*n* = 18)
Reported an increase in indoor temperature	2% (*n* = 2)

When people were asked about the use of products or any other preventive practices against mosquitoes after the HS installation, 28% (28/100) said that they stopped using other preventive practices because mosquito screening had been effective; however, most families (72%, 72/100) continued using additional measures (mainly insecticides and body repellents) because of habit or routine (33%, 25/72) or because they used those products outdoors (49%, 37/72).

The perception of an ‘increase of temperature’ associated with HS was not noticeably raised, and temperature within the house was more related to weather conditions rather to HS. Most participants did not acknowledge any increase in indoor temperature attributable to the HS (80%, 80/100) and only 18% (18/100) reported a slight overheating associated with specific hours of the day (e.g. mid‐day).

## DISCUSSION

Based on the entomological risk (presence and abundance of *Ae*. *aegypti* females) and a proxy of ATV transmission risk (indoor *Ae*. *aegypti* females infected with DENV and ZIKV), this study provides a quantitative analysis of the public health value of HS in endemic and high‐risk settings. A house protected with HS on doors and windows was ~50% less likely to contain *Ae*. *aegypti* females than unscreened houses but, more importantly, installing HS provided a ~ 70% reduced chance of having indoor DENV‐ or ZIKV‐infected *Ae*. *aegypti* females. These results (obtained from a well‐powered RCT involving 1,200 households) are strong evidence supporting HS as a method for the control of *Ae*. *aegypti* and ATVs in settings with simultaneous transmission of dengue, chikungunya and Zika.

A recent systematic review and meta‐analysis of randomised trials of individually applied housing interventions to prevent malaria and ATVs [16, 43] reported that home environmental interventions (including physical and chemical barriers to close eaves, doors and windows) reduced indoor *Aedes* and *Anopheles* densities (pooled OR = 0.35; 95% CI = 0.23 to 0.54; *p *< 0.001). Although the review did not include any intervention using house ‐screening with regular mesh for *Ae*. *aegypti*, it did include studies with insecticide‐treated house screening (ITS) – as a physical and chemical barrier – carried out by our research group in Mexico [17, 18]. These studies with ITS reported significantly fewer infestations and fewer adult *Ae*. *aegypti* females, with efficacy ranging around 60% reduction in both indices in ITS houses compared to the control. The most recent study from Merida showed that houses with ITS had ~80% less chance of having indoor *Ae*. *aegypti* females infected with ZIKV than houses without insecticidal screens [21]. Such values are ~10% higher than what we observed with HS and may be explained by the addition of insecticidal effect to the netting.

One of the important aspects of this trial has been the choice of entomological end points. Both indoor adult *Ae*. *aegypti* density and ATV infection in mosquitoes are considered the closest entomological measures to transmission risk [44, 45]. The detection of positive houses in 10‐minute sampling rounds with Prokopack aspirators has shown high levels of sensitivity for *Ae*. *aegypti* females (78.5%) and for blood‐fed females (73.3%) [36]. Similarly, such collections are sensitive at detecting ATV‐infected female *Ae*. *aegypti* [40]. Our findings, albeit entomological, provide a rigorous estimate of proxies for epidemiological measures of virus transmission.

The concept of DENV vector control does not exclusively rely on killing mosquitoes but also in reducing mosquito–human contacts as a way of decreasing or preventing virus transmission (WHO 2017, [46]. Screening entry points of a house to prevent the access of endophilic and endophagic mosquitoes – such as *Ae*. *aegypti* females – is expected to decrease the number of vectors, human exposure to infective mosquito bites and, therefore, reduce DENV, CHIKV and ZIKV transmission [7, 15, 47, 48]. If the primary household activities occur indoors, as observed in Merida, this reduced human‐mosquito contact can lead to an important epidemiological effect.

‘Mosquito‐proofing’ houses or ‘house‐screening against mosquitoes’ [9, 49] is one of the oldest methods for mosquito control, and its potential as a sustainable and effective tool for malaria control has been evaluated in randomised controlled trials [50,51]. Studies on *Anopheles* mosquitoes have showed that screened houses (screening eaves and/or doors and windows) had a 60% lower malaria prevalence than control houses without screening [52, 53, 54]. Although, ‘total’ mosquito proofing is not achieved in all cases, here we demonstrated that the number of female mosquitoes and blood‐fed females was dramatically reduced or even eliminated inside HS households. The lack of complete suppression of indoors *Ae*. *aegypti* by HS could be because this intervention does not directly reduce outdoor abundance of mosquitoes, it just prevents the entrance of mosquitoes into the house. Daily behavioural practices may likely contribute to indoor presence of mosquitoes, for example, doors of protected houses are opened every time someone enters or exits the house, providing ease of entry of mosquitoes [21, 55]. Although the ability of *Ae*. *aegypti* to breed around human habitats is considered an important risk factor for transmission of ATVs [44, 56, 57], because of the nature of the intervention our results were mainly driven by the collection of adult *Ae*. *aegypti* mosquitoes, which unfortunately left the peridomestic areas of the enrolled households out of immature‐based entomological information.

Over a decade of collaboration with the Mexican MoH, our team has generated evidence from multiple CRCTs evaluating ‘*Aedes aegypti*‐proof houses’ on entomological endpoints [7, 17, 18, 20, 21, 22]. This scientific evidence has influenced public health policies in Mexico by issuing the Official Mexican Normative for vector control (NOM‐032‐SSA2‐2014) [58] of the MoH promoting the installation of mosquito nets (with or without insecticide) on doors and windows to prevent the access of *Ae*. *aegypti* – for the prevention of DENV, CHIKV and ZIKV. While no information on the epidemiological impact of HS on human incidence of ATVs has been generated in Merida, a systematic review by Bowman et al. [15] found that HS is the best evidence‐based method supporting effectiveness in reducing DENV risk (OR 0.22, 95% CI 0.05–0.93, *p* = 0.04) after cross‐sectional and case–control studies in Australia [59, 60] and a case–control study in Taiwan [61]. Such evidence was complemented by observational studies finding that HS was protective against the risk of dengue [62, 63]. Here, our findings are encouraging as the circulation of ATVs in the mosquitoes was reduced by installing HS,however, an important limitation of our study is the lack of evidence of epidemiological impact of HS on ATVs in the human population (e.g. active surveillance of cases, seroprevalence in the exposed population, etc.). Conducting epidemiological or clinical trials for the evaluation of HS on epidemiological end points would be costly and complex to execute and to scale up; however, existing evidence (complemented by our findings) provides support for the important public health value of this approach.

Urban improvements that reduce disease vectors should be seen as an important component of many UN Sustainable Development Goals (SDGs), beyond SDG3 ‘healthy lives and well‐being’ [34, 65,66]. For example, housing and urban improvements should be aligned with SDG11 to ‘make cities and human settlements inclusive, safe, resilient and sustainable’ through improvements in the housing and basic services. Certainly, the ‘construction against *Ae*. *aegypti*’ will require close collaboration between governments, the private sector and civil society as expressed in SDG 17, which calls for ‘sustainable development through global partnerships’. Braks et al. [64], in the context of Integrated Vector Management (IVM) for Dengue control, identified at least eight SDGs and targets related to prevention of dengue. Therefore, the implementation of HS and other environmental management approaches should be out of the unique competence and economical support of a MoH. For example, in Mexico, the promotion of ‘safe housing’ with mosquito nets in doors and windows is a strategy for IVM supported by the MoH [58], but its implementation by the national vector control programme of the MoH has not been accomplished yet. In Yucatan, the Ministry of Health also recommends the installation of mosquito screens in the houses, among other preventive methods against mosquitoes. However, there are no official programmes that support, neither technically nor financially, this approach. Nevertheless, it has been emphasised that MoHs must act as stewards in other sectors to ensure that health objectives are considered in their policies [67, 68]. This includes advocating to promote access to social housing for vulnerable groups, ensuring standards for housing and empowering vulnerable groups to enhance their security and ownership.

In Mexico, HS installation is usually done professionally with high‐quality materials (such as aluminium frames) by small private companies called ‘aluminium & screens‐business’ (A&S). The current cost for protecting a house (two doors and seven windows) with HS installed by a professional is ~$ 140 USD, with the potential for sustainable impact and cost‐effectiveness after several years [18, 21, 69, 70]. Although the installation of HS was well accepted by the community and supported by the perceived reduction in mosquito abundance and biting events inside HS houses, one main limitation of HS stands on the inherent cost of materials (e.g. aluminium) and installation of the screens. People interviewed in this study mentioned cost as a major limitation to have HS. The majority receive a monthly salary that amounts to ca. 3800 pesos (≈ 190 USD) [71]. Therefore, it is understood that 70% of the responses referred the up‐front cost of the mosquito net as an impediment to its installation. While it is conceivable that a family with a minimum wage salary cannot afford to spend >70% of their monthly living budget to install HS, offering mechanisms to micro‐credit or other ways of reducing the up‐front cost of HS may lead to more uptake of this intervention. Another immediate solution to increase community access and make HS more affordable is introducing certain cost‐saving strategies, that is, the use of less‐expensive materials rather than aluminium frames, within the list of options offered by A&S businesses or do‐it‐yourself (DIY) for instance, already made and ready‐to‐install mosquito screens. Our team, with support of IDRC, is currently developing studies which include the evaluation of different DIY options for the protection of doors and windows, to replace aluminium frames and professional installation and ultimately, to enhance community access to house screening and promote the participation/engagement of the small business sector to improve *Ae*. *aegypti* control.

Perhaps one of the paths for mass implementation of HS is to reframe it as a public health good, which would allow involving distinctive administrative and legal atmospheres, from central government, state departments and regional or local authorities (municipalities) [16]. The interlinkages between housing and health can serve as a starting point for MoHs to work with other ministries to initiate policy processes to improve national and local housing standards. For example, the concept and practice of ‘safe housing’ from the MoH in Mexico could unite public health with those of other homonymous and/or related programmes for ‘safe housing’ already in place within the Mexican National Program of housing [72], SEDATU, CONAVI and INFONAVIT, which already tries to incorporate the seven elements of adequate housing established by UN‐Habitat, for example, security of tenancy,availability of services, materials, facilities and infrastructure; affordability, habitability and accessibility; location and cultural adequacy. It will be important to call attention to health as an important component in addition to safety and dignity as part of the concept of habitability. The health impact of HS and other house improvements can go far beyond to include decreased indoor mosquito density to reduce and prevent mosquito‐borne and other infectious diseases. The interventions might possibly translate into substantial improvements in morbidity, mortality and family health as well as social and economic impact attributable to vector‐borne diseases.

In conclusion, the significant impact of HS on populations of the primary vector of DENV, CHIKV, ZIKV, yellow fever virus (YFV) and Mayaro viruses (MYV) provides good evidence for HS to be considered as an important strategy for integrated vector management approaches in ATVs endemic countries and territories. In this study, we observed a reduction in the number of indoor *Ae*. *aegypti* mosquitoes, which was reflected in lower mosquito infection rates of important human arboviruses such as DENV and ZIKV. These results along with our positive evidence of good social acceptance for HS among the targeted communities suggest that HS could impact the incidence of arboviral diseases during seasonal transmission in endemic areas, although clinical trials are still warranted to quantify its epidemiological impact.

## Supporting information

Fig S1

Table S1‐S4

## References

[1] PAHO . 2021. Dengue. Available from: https://www.paho.org/en/topics/dengue.

[2] Yactayo S , Staples JE , Millot V , Cibrelus L , Ramon‐Pardo P . Epidemiology of chikungunya in the Americas. J Infect Dis. 2016;214:S441–5. 27920170 10.1093/infdis/jiw390 PMC5137246

[3] PAHO . 2017. Pan‐American Health Organization. Zika ‐ Actualización Epidemiológica Regional de la OPS (Américas) 25 de agosto de 2017. Available from: https://www3.paho.org/hq/index.php?option=com_content&view=article&id=11599:regional‐zika‐epidemiological‐update‐americas&Itemid=41691&lang=es.

[4] Girard M , Nelson CB , Picot V , Gubler DJ . Arboviruses: a global public health threat. Vaccine. 2020;38(24):3989–94. 32336601 10.1016/j.vaccine.2020.04.011 PMC7180381

[5] Gubler DJ . Dengue, urbanization and globalization: the unholy trinity of the 21(st) century. Trop Med Health. 2011;39(4 Suppl):3–11. 10.2149/tmh.2011-S05 PMC3317603 22500131

[6] World Health Organization . Keeping the vector out: housing improvements for vector control and sustainable development. Geneva: World Health Organization; 2017. Licence: CC BY‐NC‐SA 3.0 IGO., WHO, editor. Geneva: 2017. https://apps.who.int/iris/handle/10665/259404.

[7] Vazquez‐Prokopec G , Lenhart A , Manrique‐Saide P . Housing Improvement: a renewed paradigm for urban vector‐borne disease control? Trans R Soc Trop Med Hyg. 2016:110(10):567–9. 27864518 10.1093/trstmh/trw070

[8] Wilson AL , Courtenay O , Kelly‐Hope LA , Scott TW , Takken W , Torr SJ , et al. The importance of vector control for the control and elimination of vector‐borne diseases. PLoS Negl Trop Dis. 2020;14(1):e0007831. 31945061 10.1371/journal.pntd.0007831 PMC6964823

[9] Lindsay SW , Emerson PM , Charlwood JD . Reducing malaria by mosquito‐proofing houses. Trends Parasitol. 2002;18(11):510–4. 12473368 10.1016/s1471-4922(02)02382-6

[10] Horstick O , Runge‐Ranzinger S . Protection of the house against Chagas disease, dengue, leishmaniasis, and lymphatic filariasis: a systematic review. Lancet Infect Dis. 2018;18(5):e147–58. 10.1016/S1473-3099(17)30422-X 29074038

[11] Lindsay S , Wilson A , Golding N , Scott TW , Takken W . Improving the built environment in urban areas to control *Aedes aegypti*‐borne diseases. Bull. WHO. 2017;95:607–8. 28804174 10.2471/BLT.16.189688 PMC5537749

[12] World Health Organization . Handbook for Integrated Vector Management. World Health Organization 2012. ISBN 978 92 4150280. Available from: http://apps.who.int/iris/bitstream/10665/44768/1/9789241502801_eng.pdf

[13] World Health Organization . Global vector control response 2017–2030. Global vector control response 2017–2030.World Health Organization. (2009). Dengue guidelines for diagnosis, treatment, prevention and control, new edn. Geneva: World Health Organization; 2017. http://www.who.int/iris/handle/10665/44188

[14] Olliaro P , Fouque F , Kroeger A , Bowman L , Velayudhan R , Santelli AC , et al. Improved tools and strategies for the prevention and control of arboviral diseases: a research‐to‐policy forum. PLoS Negl Trop Dis. 2018;12(2):e0005967. 29389959 10.1371/journal.pntd.0005967 PMC5794069

[15] Bowman LR , Donegan S , McCall PJ . Is dengue vector control deficient in effectiveness or evidence?: Systematic review and meta‐analysis. PLoS Negl Trop Dis. 2016;10(3):e0004551. 26986468 10.1371/journal.pntd.0004551 PMC4795802

[16] Kua KP , Lee SWH . Randomized trials of housing interventions to prevent malaria and Aedes‐transmitted diseases: a systematic review and meta‐analysis. PLoS One. 2021;16(1):e0244284. 33417600 10.1371/journal.pone.0244284 PMC7793286

[17] Che‐Mendoza A , Guillermo‐May G , Herrera‐Bojórquez J , Barrera‐Pérez M , Dzul‐Manzanilla F , Gutierrez‐Castro C , et al. Long‐lasting insecticide treated house screens and targeted treatment of productive breeding‐sites for dengue vector control in Acapulco, Mexico. Trans R Soc Trop Med Hyg. 2015;109(2):106–15. 25604761 10.1093/trstmh/tru189 PMC4299524

[18] Che‐Mendoza A , Medina‐Barreiro A , Koyoc‐Cardeña E , Uc‐Puc V , Contreras‐Perera Y , Herrera‐Bojórquez J , et al. House screening with insecticide‐treated netting provides sustained reductions in domestic populations of *Aedes aegypti* in Merida, Mexico. PLoS Negl Trop Dis. 2018;12(3):e0006283. 29543805 10.1371/journal.pntd.0006283 PMC5870999

[19] Herrera‐Bojórquez J , Trujillo‐Peña E , Vadillo Sánchez J , Riestra‐Morales M , Che‐Mendoza A , Delfín‐González H , et al. Efficacy of long‐lasting insecticidal nets with declining physical and chemical integrity on *Aedes aegypti* . J Med Entomol. 2020;57(2):503–10. 31603517 10.1093/jme/tjz176

[20] Manrique‐Saide P , Che‐Mendoza A , Barrera‐Pérez M , Guillermo‐May G , Herrera Bojorquez J , Dzul‐Manzanilla F , et al. Use of insecticide‐treated house screens to reduce infestations of dengue virus vectors, Mexico. Emerg Infect Dis. 2015;21(2):308–11. 25625483 10.3201/eid2102.140533 PMC4313634

[21] Manrique‐Saide P , Herrera‐Bojórquez J , Medina‐Barreiro A , Trujillo‐Peña E , Villegas‐Chim J , Valadez‐González N , et al. Insecticide‐treated house screening protects against Zika‐infected *Aedes aegypti* in Merida, Mexico. PLoS Negl Trop Dis. 2021;15(1):e0009005. 33465098 10.1371/journal.pntd.0009005 PMC7853519

[22] Jones C , Benítez‐Valladares D , Barrera‐Pérez M , Selem‐Salas C , Chablé‐Santos J , Dzul‐Manzanilla F , et al. Use and acceptance of Long‐Lasting Insecticidal Nets for dengue prevention in Acapulco, Guerrero, Mexico. BMC Public Health. 2014;14(1):846. 25124670 10.1186/1471-2458-14-846 PMC4152567

[23] CENAPRECE 2020 . Lista_de_Insumos_Recomendados_por_el_CENAPRECE. Available from:https://www.gob.mx/cms/uploads/attachment/file/469289/Lista_de_Insumos_Recomendados_por_el_CENAPRECE.pdf.

[24] COESPO . Catálogo sistema urbano nacional. 2012. COESPO. Available from: http://coespo.yucatan.gob.mx/general/Sietema_urbano_naciona_Catalogo_2012.pdf.

[25] Che‐Mendoza A , Martin‐Park A , Chávez‐Trava JM , Contreras‐Perera Y , Delfín‐González H , González‐Olvera G , et al. Abundance and seasonality of *Aedes aegypti* (Diptera: Culicidae) in two suburban localities of South Mexico, with implications for Wolbachia (Rickettsiales: Rickettsiaceae)‐carrying male releases for population suppression. J Med Entomol. 2021;2:tjab052. 10.1093/jme/tjab052 PMC8285091 33822117

[26] Dzul‐Manzanilla F , Correa‐Morales F , Che‐Mendoza A , Palacio‐Vargas J , Sánchez‐Tejeda G , González‐Roldan JF , et al. Identifying urban hotspots of dengue, chikungunya and Zika transmission in Mexico to support risk stratification efforts. Lancet Planetary Health. 2021;5:e277–85. 33964237 10.1016/S2542-5196(21)00030-9 PMC8114339

[27] Bisanzio D , Dzul‐Manzanilla F , Gomez‐Dantés H , Pavia‐Ruz N , Hladish TJ , Lenhart A , et al. Spatio‐temporal coherence of dengue, chikungunya and Zika outbreaks in Merida, Mexico. Plos Negl Trop Dis. 2018;12:e0006298. 29543910 10.1371/journal.pntd.0006298 PMC5870998

[28] SINAVE . 2020. BoletínEpidemiológico Sistema Nacional de Vigilancia Epidemiológica Sistema Único de Información. Available from: https://www.gob.mx/salud/acciones‐y‐programas/direccion‐general‐de‐epidemiologia‐boletin‐epidemiologico.

[29] Lloyd LS , Winch P , Ortega‐Canto J , Kendall C . Results of a community‐based *Aedes aegypti* control program in Merida, Yucatan, Mexico. Am J Trop Med Hyg. 1992;46:635–42. 1621887 10.4269/ajtmh.1992.46.635

[30] Manrique‐Saide P , Davies CR , Coleman PG , Rebollar‐Tellez E , Che‐Medoza A , Dzul‐Manzanilla F , et al. Pupal Surveys for *Aedes aegypti*: Surveillance and potential targeted control in residential areas of Mérida, México. J Am Mosq Control Assoc. 2008;24:289–98. 18666538 10.2987/5578.1

[31] Manrique‐Saide P , Coleman P , McCall PJ , Lenhart A , Vázquez‐Prokopec G , Davies CR . Multi‐scale analysis of the associations among egg, larval and pupal surveys and the presence and abundance of adult female *Aedes aegypti* (*Stegomyia aegypti*) in the city of Merida, Mexico. Med Vet Entomol. 2014;28(3):264–72. 24797405 10.1111/mve.12046

[32] García‐Rejón JE , López‐Uribe MP , Loroño‐Pino MA , Farfán‐Ale JA , Najera‐Vazquez MR , Lozano‐Fuentes S , et al. Productive container types for *Aedes aegypti* immatures in Mérida, México. J Med Entomol. 2011;48:644–50. 21661326 10.1603/me10253

[33] Winch PJ , Barrientos‐Sanchez G , Puigserver‐Castro E , Manzano‐Cabrera L , Lloyd LS , Mendez‐Galvan JF . Variation in *Aedes aegypti* larval indices over a one‐year period in a neighborhood of Merida, Yucatan, Mexico. J Am Mosq Control Assoc. 1992;8:193–5. 1431864

[34] Manrique‐Saide P , Uc V , Prado C , Carmona C , Vadillo J , Chan R , et al. Storm sewers as larval habitats for *Aedes aegypti* and Culex spp. in a neighborhood of Merida, Mexico. J Am Mosq Control Assoc. 2012;28:255–7. 23833907 10.2987/12-6244R.1

[35] Manrique‐Saide P , Arisqueta‐Chable C , Geded‐Moreno E , Herrera‐Bojórquez J , Uc V , Chable‐Santos J , et al. An assessment of the importance of subsurface catch basins for *Aedes aegypti* adult production during the dry season in a neighbourhood of Merida, Mexico. J Am Mosq Control Assoc. 2013;29:164–7. 23923331 10.2987/12-6320R.1

[36] Koyoc‐Cardeña E , Medina‐Barreiro A , Cohuo‐Rodríguez A , Pavía‐Ruz N , Lenhart A , Ayora‐Talavera G , et al. Estimating absolute indoor density of *Aedes aegypti* (Stegomyia) using removal sampling. Parasites Vectors. 2019;12(1):250. 31113454 10.1186/s13071-019-3503-y PMC6528352

[37] Hernandez‐Avila JE , Rodriguez M‐H , Santos‐Luna R , Sanchez‐Castañeda V , Roman‐Perez S , Rios‐Salgado VH , et al. Nation‐wide, web‐based, geographic information system for the integrated surveillance and control of dengue fever in Mexico. PLoS One. 2013;8:e70231. 23936394 10.1371/journal.pone.0070231 PMC3735575

[38] Vazquez‐Prokopec GM , Galvin WA , Kelly R , Kitron U . A new, cost‐effective, battery‐powered aspirator for adult mosquito collections. J Med Entomol. 2009;46(6):1256–9. 19960668 10.1603/033.046.0602 PMC2800949

[39] Puerta‐Guardo H , Contreras‐Perera Y , Perez‐Carrillo S , Che‐Mendoza A , Ayora‐Talavera G , Vazquez‐Prokopec G , et al. UCBE‐LCB Team, Wolbachia in native populations of Aedes albopictus (Diptera: Culicidae) From Yucatan Peninsula, Mexico. Journal of Insect Science. 2020;20(5):1–7. 10.1093/jisesa/ieaa096 PMC7583270 33034342

[40] Ahmad NA , Mancini M‐V , Ant TH , Martinez J , Kamarul GMR , Nazni WA , et al. Wolbachia strain wAlbB maintains high density and dengue inhibition following introduction into a field population of *Aedes aegypti* . Phil Trans R Soc B. 2021;376:20190809. 33357050 10.1098/rstb.2019.0809 PMC7776933

[41] Lanciotti RS , Kosoy OL , Laven JJ , Velez JO , Lambert AJ , Johnson AJ , et al. Genetic and serologic properties of Zika virus associated with an epidemic, Yap State, Micronesia, 2007. Emerg Infect Dis. 2008;14(8):1232–9. 18680646 10.3201/eid1408.080287 PMC2600394

[42] Halloran ME , Longini IM , Struchiner CJ . Design and analysis of vaccine studies. New York: Springer Verlag; 2010.

[43] Buhler C , Winkler V , Runge‐Ranzinger S , Boyce R , Horstick O . Environmental methods for dengue vector control – a systematic review and meta‐analysis. PLoS Negl Trop Dis. 2019;13(7):e0007420. 31295250 10.1371/journal.pntd.0007420 PMC6650086

[44] Kirstein O , Ayora‐Talavera G , Koyoc‐Cardeña E , Chan‐Espinoza D , Che‐Mendoza A , Cohuo‐Rodriguez A , et al. Natural arbovirus Infection Rate and Detectability in indoor female *Aedes aegypti*, Merida, Yucatan, Mexico. PLoS Negl Trop Dis. 2021;15(1):e0008972. 33395435 10.1371/journal.pntd.0008972 PMC7781390

[45] Scott TW , Morrison AC . Vector dynamics and transmission of dengue virus: Implications for dengue surveillance and prevention strategies: vector dynamics and dengue prevention. In: Rothman AL , editor. Dengue Virus. Berlin: Springer‐Verlag, Berlin; 2010. pp. 115–28. 10.1007/978-3-642-02215-9_9 19802582

[46] Ritchie S , Devine G , Vazquez‐Prokopec G , Lenhart A , Manrique‐Saide P , Scott TW . Insecticide‐based approaches for dengue vector control. In: Koenraadt C , Spitzen J , Takken W , editors. Innovative Strategies for Vector Control. Wageningen Academic Publishers; 2021.

[47] Roiz D , Wilson AL , Scott TW , Fonseca DM , Jourdain F , Müller P , et al. Integrated Aedes management for the control of Aedes‐borne diseases. PLoS Negl Trop Dis. 2018;12(12):e0006845. 30521524 10.1371/journal.pntd.0006845 PMC6283470

[48] Wilson AL , Dhiman RC , Kitron U , Scott TW , van den Berg H , Lindsay SW . Benefit of insecticide‐treated nets, curtains and screening on vector borne diseases, excluding malaria: a systematic review and meta‐analysis. PLoS Negl Trop Dis. 2014;8(10):e3228. 25299481 10.1371/journal.pntd.0003228 PMC4191944

[49] Walker N . The hygienic house: mosquito‐proofing with screens. Am J Trop Med Hyg. 2010;83(5):963–4. 21036821 10.4269/ajtmh.2010.10-0405 PMC2963953

[50] Kirby MJ , Ameh D , Bottomley C , Green C , Jawara M , Milligan PJ , et al. Effect of two different house screening interventions on exposure to malaria vectors and on anaemia in children in The Gambia: a randomised controlled trial. Lancet. 2009;374:998–1009. 19732949 10.1016/S0140-6736(09)60871-0 PMC3776946

[51] Tusting LS , Ippolito MM , Willey BA , et al. The evidence for improving housing to reduce malaria: a systematic review and meta‐analysis. Malar J. 2015;14:209. 26055986 10.1186/s12936-015-0724-1 PMC4460721

[52] Bradley J , Rehman AM , Schwabe C , Vargas D , Monti F , Ela C , et al. Reduced prevalence of malaria infection in children living in houses with window screening or closed eaves on Bioko Island, Equatorial Guinea. PLoS One. 2013;8(11):e80626. 24236191 10.1371/journal.pone.0080626 PMC3827414

[53] Getawen SK , Ashine T , Massebo F , Woldeyes D , Lindtjørn B . Exploring the impact of house screening intervention on entomological indices and incidence of malaria in Arba Minch town, southwest Ethiopia: a randomized control trial. Acta Trop. 2018;181:84–94. 29452110 10.1016/j.actatropica.2018.02.009

[54] Ng'ang'a PN , Okoyo C , Mbogo C , Mutero CM . Evaluating effectiveness of screening house eaves as a potential intervention for reducing indoor vector densities and malaria prevalence in Nyabondo, western Kenya. Malar J. 2020;19(1):341. 32950061 10.1186/s12936-020-03413-3 PMC7501660

[55] Dzul‐Manzanilla F , Ibarra‐López J , Bibiano Marín W , Martini‐Jaimes A , Leyva JT , Correa‐Morales F , et al. Indoor resting behavior of *Aedes aegypti* (Diptera: Culicidae) in Acapulco, Mexico. J Med Entomol. 2017;54(2):501–4. 28011725 10.1093/jme/tjw203

[56] Scott TW , Morrison AC . *Aedes aegypti* density and the risk of dengue‐virus. Ecological Aspects for Application of Genetically Modified Mosquitoes. 2003;2:187.

[57] Esu E , Lenhart A , Smith L , Horstick O . Effectiveness of peridomestic space spraying with insecticide on dengue transmission; systematic review. Trop Med Int Health. 2010;15(5):619–31. 20214764 10.1111/j.1365-3156.2010.02489.x

[58] Diario Oficial de la Federación (DOF) . NORMA Oficial Mexicana NOM‐032‐SSA2‐2014, Para la vigilancia epidemiológica, promoción, prevención y control de las enfermedades transmitidas por vectores. 2015. Available from: https://www.dof.gob.mx/nota_detalle.php?codigo=5389045&fecha=16/04/2015.

[59] McBride WJ , Mullner H , Muller R , Labrooy J , Wronski I . Determinants of dengue 2 infection among residents of Charters Towers, Queensland, Australia. Am J Epidemiol. 1998;148(11):1111–6. 9850134 10.1093/oxfordjournals.aje.a009589

[60] Murray‐Smith S , Weinstein P , Skelly C . Field epidemiology of an outbreak of dengue fever in Charters Towers, Queensland: are insect screens protective? Aust New Zea J Pub Health. 1996;20(5):545–7. 10.1111/j.1467-842x.1996.tb01637.x 8987228

[61] Ko YC , Chen MJ , Yeh SM . The predisposing and protective factors against dengue virus transmission by mosquito vector. Am J Epidemiol. 1992;136(2):214–20. 1415143 10.1093/oxfordjournals.aje.a116487

[62] Halstead S , Gómez‐Dantés H . Dengue a worldwide problem, a common strategy. Mexican Ministry of Health‐Rockefeller Foundation. Mexico, D.F. 1992.

[63] Koopman JS , Prevots DR , Vaca Marin MA , Gomez Dantes H , Zarate Aquino ML , Longini IM Jr , et al. Determinants and predictors of dengue infection in Mexico. Am J Epidemiol. 1991;133(11):1168–78. 2035520 10.1093/oxfordjournals.aje.a115829

[64] Braks M , Giglio G , Tomassone L , Sprong H , Leslie T . Making vector‐borne disease surveillance work: new opportunities from the SDG perspectives. Front Vet Sci. 2019;16(6):232. 10.3389/fvets.2019.00232 PMC6647909 31380399

[65] Tusting LS , Cairncross S , Ludolph R , Velayudhan R , Wilson AL , Lindsay SW . Assessing the health benefits of development interventions. BMJ Glob Health. 2021;6(2):e005169. 10.1136/bmjgh-2021-005169 PMC7888316 33593756

[66] Horstick O , Runge‐Ranzinger S . Multisectoral approaches for the control of vector‐borne diseases, with particular emphasis on dengue and housing. Trans R Soc Trop Med Hyg. 2019;113(12):823–8. 31034038 10.1093/trstmh/trz020

[67] Figueras J , McKee M , editors. Health systems, health, wealth and societal well‐being: assessing the case for investing in health systems. Maidenhead, UK: Open University Press; 2012.

[68] World Health Organization . Housing and Health Guidelines. Geneva: World Health Organization; 2018. Available from: https://www.ncbi.nlm.nih.gov/books/NBK535293/

[69] Alfonso‐Sierra E , Basso C , Beltrán‐Ayala E , Mitchell‐Foster K , Quintero J , Cortés S , et al. Innovative dengue vector control interventions in Latin America: what do they cost? Pathog Glob Health. 2016;110(1):14–24. 26924235 10.1080/20477724.2016.1142057 PMC4870030

[70] Quintero J , Brochero H , Manrique‐Saide P , Barrera‐Perez M , Basso C , Romero S , et al. Ecological, biological and social dimensions of dengue vector breeding in five urban settings of Latin America: a multi‐country study. BMC Infect Dis. 2014;14:38. 24447796 10.1186/1471-2334-14-38 PMC3904013

[71] CONASAMI . Comsion Nacional de Salarios Minimos. 2021. Available from: https://www.gob.mx/conasami.

[72] SEDATU . Secretaria de Desarrollo agrario, territorial y urbano. 2019. Available from: https://www.cmic.org.mx/comisiones/sectoriales/vivienda/2020/SEDATU/PNV/Programa_Nacional_de_Vivienda_2019‐2024.pdf

