# Supplementary material for: Protective effect of house screening against indoor Aedes aegypti in Mérida, Mexico: A cluster randomised controlled trial
Source: Trop Med Int Health. 2021 Oct 21;26(12):1677–88. doi: 10.1111/tmi.13680 (PMC9298035; doi:10.1111/tmi.13680)
Supplement: Supplementary file 1 — Fig S1 [file TMI-26-1677-s002.docx]

**Supplementary Figure 1.** Flow diagram for households enrolled in of the cluster randomized controlled trial of house-screening against indoor *Aedes aegypti*, Mexico.

Assessed for eligibility

(n=12 clusters)

(n=100 households per cluster)

(n= 1,200 houses)

## Enrollment

## Allocation clusters

Entomological survey (n=180 houses)

Social assessment (n=100 houses)

ARB-molecular virology (n=80 houses)

Lost to follow-up (give reasons) (n= 0)

Discontinued intervention (give reasons) (n= 0)

Entomological survey (n=180 houses)

Social assessment (n=100 houses)

ARB-molecular virology (n= 103 houses)

Lost to follow-up (give reasons) (n= 0)

Discontinued intervention (give reasons) (n= 0)

## Post-intervention Entomology

Randomized [n= 30 houses per cluster (n= 12) = 360 houses)

Analysed (n= 180)
♦ Excluded from analysis (give reasons) (n= 0)

Analysed (n=180)
♦ Excluded from analysis (give reasons) (n= 0)

## Analysis

## Baseline Entomology

Group B = Control

Group A = Treatment

(HS)

## Randomization Treatments

Randomization into Group A (n= 6 clusters: 180 houses)

Randomization into Group B (n= 6 clusters: 180 houses)
