# Supplementary material for: Protective effect of house screening against indoor Aedes aegypti in Mérida, Mexico: A cluster randomised controlled trial
Source: Trop Med Int Health. 2021 Oct 21;26(12):1677–88. doi: 10.1111/tmi.13680 (PMC9298035; doi:10.1111/tmi.13680)
Supplement: Supplementary file 2 — Table S1‐S4 [file TMI-26-1677-s001.docx]

**Supplementary Material**

**Table S1:** Baseline of construction features of houses included per cluster.

| Variable | Category | Treatment houses (n=100) | | Control houses (n=100) | |
| --- | --- | --- | --- | --- | --- |
|  |  | n (± se) | range or % | n (± se) | range or % |
| General | *Single floor level* | 92 (0.27) | 92% | 98 (0.14) | 98% |
|  | *Front or back yard* | 91 (0.28) | 91% | 100 | 100% |
| Floor | *Mixed* | 15 (0.79) | 15% | 32 (0.47) | 32% |
|  | *Concrete* | 8 (0.78) | 8% | 66 (0.50) | 66% |
|  | *Tile* | 77 (0.76) | 77% | 2 (0.97) | 2% |
| Roof | *Mixed* | 10 (0.62) | 10% | - | - |
|  | *Concrete* | 89 (0.60) | 89% | 97 (0.20) | 97% |
|  | *Metal* | 1 (0) | 1% | 3 (0.17) | 3% |
| Walls | *Concrete* | 97 (0.17) | 97% | 100 | 100% |
|  | *Mixed* | 3 (0.46) | 3% | - | - |
| Water supply | *Piped water* | 100 (0) | 100% | 100 | 100% |
| Water storage | *Yes* | - | - | - | - |
|  | *No* | 100 (0) | 100% | 100 | 100% |
| Electricity | *Yes* | 100 (0) | 100% | 100 | 100% |
|  | *No* | - | - | - | - |
| Number of Doors | *1* | 2 | 2% | 2 (0.31) | 2% |
|  | *2* | 78 | 78% | 95 (0.20) | 95% |
|  | *3* | 18 | 18% | 3 (0.40) | 3% |
|  | *4* | 2 | 2% | - | - |
| Number of windows | *1* | 3 (2.16) | 3% | - | - |
|  | *2* | 3 (2.20) | 3% | - | - |
|  | *3* | 3 (2.28) | 3% | - | - |
|  | *4* | 22 (2.06) | 22% | - | - |
|  | *5* | 23 (1.90) | 23% | 9 (0.74) | 9% |
|  | *6* | 18 (1.97) | 18% | 34 (0.70) | 34% |
|  | *7* | 19 (2.02) | 19% | 53 (0.71) | 53% |
|  | *8* | 5 (2.01) | 5% | 4 (0.79) | 4% |
|  | *9* | 1 | 1% | - | - |
|  | *12* | 3 (3.5) | 3% | - | - |

**Table S2.** Demographic data of participants accepting the intervention and to whom KAP-survey was applied during the enrolment process, and later on (post-intervention) were interviewed for social acceptance and perceived efficacy of house-screening.

| **Profile** | **Total** | |
| --- | --- | --- |
|  | N (150) | (%) |
| **Sex** |  |  |
| Female | 118 | 79 |
| Male | 32 | 21 |
| **Occupation** |  |  |
| Housewife | 81 | 55 |
| Employee | 27 | 18 |
| Unemployed | 4 | 3 |
| Other | 35 | 24 |
| **Education** |  |  |
| Primary level | 48 | 33 |
| Secondary level | 47 | 32 |
| High School | 19 | 13 |
| Technical career | 20 | 14 |
| Bachelor’s degree | 9 | 6 |
| No studies | 4 | 3 |

**Table S3.** Main symptoms associated with dengue (DEN), chikungunya (CHIK) and Zika (ZIK) mentioned by the participants.

| **Symptoms** | **DEN** | | **CHIK** | | **ZIK** | |
| --- | --- | --- | --- | --- | --- | --- |
|  | n | (%) | n | (%) | n | (%) |
| **Headache** | 83 | 15 | 70 | 13 | 45 | 10 |
| **Joint pain** | 104 | 19 | 139 | 26 | 85 | 20 |
| **Body pain** | 95 | 17 | 83 | 16 | 58 | 14 |
| **Bleeding gums** | 25 | 5 | 17 | 3 | - | - |
| **Fever** | 127 | 23 | 99 | 19 | 67 | 16 |
| **Doesn’t know** | 13 | 2 | 28 | 5 | 70 | 17 |
| **Pruritus** | 19 | 3 | 25 | 5 | - | - |
| **Vomit** | 23 | 4 | 18 | 3 | 17 | 3 |

**Table S4. CONSORT 2010 checklist of information to include when reporting a randomized trial.**

| Section/Topic | Item No | Checklist item | Reported on page No |
| --- | --- | --- | --- |
| Title and abstract | | | |
|  | 1a | Identification as a randomized trial in the title. | 1 |
|  | 1b | Structured summary of trial design, methods, results, and conclusions (for specific guidance see CONSORT for abstracts) | 2 |
| Introduction | | | |
| Background and objectives | 2a | Scientific background and explanation of rationale | 3-4 |
|  | 2b | Specific objectives or hypotheses | 4 |
| Methods | | | |
| Trial design | 3a | Description of trial design (such as parallel, factorial) including allocation ratio | 4-6; Fig.1., Suppl. Fig. 1 |
|  | 3b | Important changes to methods after trial commencement (such as eligibility criteria), with reasons | 5-6 |
| Participants | 4a | Eligibility criteria for participants | 5-6 |
|  | 4b | Settings and locations where the data were collected | 4-5, Fig.1, Table S1. |
| Interventions | 5 | The interventions for each group with sufficient details to allow replication, including how and when they were actually administered. | 5-6 |
| Outcomes | 6a | Completely defined pre-specified primary and secondary outcome measures, including how and when they were assessed | 6-8, Table 1 & 2 |
|  | 6b | Any changes to trial outcomes after the trial commenced, with reasons | 5 |
| Sample size | 7a | How sample size was determined | 5-6 |
|  | 7b | When applicable, explanation of any interim analyses and stopping guidelines |  |
| Randomisation: |  |  |  |
| Sequence generation | 8a | Method used to generate the random allocation sequence | 5, 7 |
|  | 8b | Type of randomisation; details of any restriction (such as blocking and block size) | 5-7 |
| Allocation concealment mechanism | 9 | Mechanism used to implement the random allocation sequence (such as sequentially numbered containers), describing any steps taken to conceal the sequence until interventions were assigned | 5-7 |
| Implementation | 10 | Who generated the random allocation sequence, who enrolled participants, and who assigned participants to interventions | 17, PMS, NPR. |
| Blinding | 11a | If done, who was blinded after assignment to interventions (for example, participants, care providers, those assessing outcomes) and how | 5, 8 |
|  | 11b | If relevant, description of the similarity of interventions | 5, Table S1. |
| Statistical methods | 12a | Statistical methods used to compare groups for primary and secondary outcomes | 9 |
|  | 12b | Methods for additional analyses, such as subgroup analyses and adjusted analyses | NA |
| Results | | | |
| Participant flow (a diagram is strongly recommended) | 13a | For each group, the numbers of participants who were randomly assigned, received intended treatment, and were analyzed for the primary outcome | 5, 7 & Suppl. Fig. 1 |
|  | 13b | For each group, losses and exclusions after randomisation, together with reasons | 5, 7 & Suppl. Fig, 1 |
| Recruitment | 14a | Dates defining the periods of recruitment and follow-up | 5-7 |
|  | 14b | Why the trial ended or was stopped | 5 |
| Baseline data | 15 | A table showing baseline demographic and clinical characteristics for each group | Table S1, S2 & S3. |
| Numbers analysed | 16 | For each group, number of participants (denominator) included in each analysis and whether the analysis was by original assigned groups | 5 & Suppl. Fig, 1 |
| Outcomes and estimation | 17a | For each primary and secondary outcome, results for each group, and the estimated effect size and its precision (such as 95% confidence interval) | Table 1. |
|  | 17b | For binary outcomes, presentation of both absolute and relative effect sizes is recommended |  |
| Ancillary analyses | 18 | Results of any other analyses performed, including subgroup analyses and adjusted analyses, distinguishing pre-specified from exploratory | NA |
| Harms | 19 | All-important harms or unintended effects in each group (for specific guidance see CONSORT for harms) | NA |
| Discussion | | | |
| Limitations | 20 | Trial limitations, addressing sources of potential bias, imprecision, and, if relevant, multiplicity of analyses | Discussion section (12-16). |
| Generalisability | 21 | Generalisability (external validity, applicability) of the trial findings | 15-16 |
| Interpretation | 22 | Interpretation consistent with results, balancing benefits and harms, and considering other relevant evidence | 13-16 |
| Other information | | |  |
| Registration | 23 | Registration number and name of trial registry | NA |
| Protocol | 24 | Where the full trial protocol can be accessed, if available | Available upon request |
| Funding | 25 | Sources of funding and other support (such as supply of drugs), role of funders | 15 |

NA: Not applicable.
